# Challenges in Standardizing 3D Echocardiographic Measurements: The Need for Universal Guidelines

**DOI:** 10.31661/gmj.v13i.3564

**Published:** 2024-09-13

**Authors:** Alaaldin Hoshmand, Elnaz Javanshir

**Affiliations:** ^1^ Cardiovascular Research Center, Tabriz University of Medical Sciences, Tabriz, Iran

**Keywords:** 3D Echocardiography, eart Ventricles, Image Processing, tandardization, Cardiovascular Imaging, Clinical Protocols

## Dear Editor,

**Table:** Table1. Summary of Current Standards Compared to Proposed Universal Guidelines for 3D Echocardiographic Measurements

**Measurement Parameter**	**Current Standard**	**Proposed Universal Guideline**	**Variability**	**References**
Left Ventricular Volume	2D echocardiography; manual tracing methods	Automated 3D echocardiography; standardized algorithms with minimal manual correction	High variability due to operator dependence and manual tracing errors	[2][5]
Ejection Fraction	2D echocardiography; volume derived; geometric assumptions	3D echocardiography; direct volume measurements; automated algorithms	Moderate variability due to geometric assumptions in 2D methods	[2][6]
Right Ventricular Function	2D echocardiography; TAPSE and fractional area change	3D echocardiography; full-volume assessment	High variability due to dependence on operator skill and image quality	[3][6]
Atrial Volume	2D echocardiography; area-length method	3D echocardiography; direct measurement with atrial-focused views	Moderate variability due to atrial foreshortening and operator technique	[7]
Global Longitudinal Strain	2D speckle-tracking echocardiography	Standardized 3D speckle-tracking echocardiography	High variability due to vendor-specific software differences	[8]
Mitral Valve Area (Planimetry)	2D echocardiography; geometric assumptions	3D echocardiography; Planimetry by 3D multiplanar reconstruction (MPR) with real-time 3D imaging	Moderate variability due to reliance on geometric assumptions in 2D	[9][10]

As echocardiography continues to evolve with technological advancements, three-dimensional echocardiography (3DE) has emerged as a powerful tool, offering enhanced accuracy and reproducibility in cardiac imaging [1]. However, despite its potential, the standardization of 3D echocardiographic measurements remains a significant challenge in clinical practice [2]. This letter seeks to point out major challenges to the standardization of 3D echocardiographic measurements and, more importantly, call for universal guidelines in that aspect to ensure consistency and reliability across different institutions.

The adoption of 3DE in routine clinical practice has been slow, largely due to the lack of standardization in measurement techniques and interpretation [3]. Traditional two-dimensional echocardiography (2DE) has long been the standard, but it is limited by geometric assumptions and variability in operator technique [4]. In contrast, 3DE provides direct volume measurements, which can potentially eliminate these limitations [5]. However, the variability in 3DE measurements remains high, mainly due to differences in image acquisition, post-processing techniques, and the experience level of the operators [2]. Table-1 highlights the need for universal guidelines in standardizing 3D echocardiographic measurements to reduce variability and improve clinical outcomes.

Initial challenges in standardizing 3DE measurements is the dependence on operator skill and the quality of the equipment used [11]. Studies have shown that automated 3DE analysis can yield accurate and reproducible measurements of left ventricular (LV) volumes and ejection fraction (EF) when performed by experienced operators using high-quality images [12]. However, in less experienced hands or with suboptimal image quality, the variability in measurements can be significant, undermining the reliability of 3DE in clinical practice [3].

Another issue is the lack of consensus on the best practices for image acquisition and post-processing [8]. Different vendors offer varying software solutions for 3DE analysis, each with unique algorithms and default settings. This vendor-specific variability contributes to inconsistencies in measurements, making it difficult to compare results across different institutions or even within the same institution over time [13].

Furthermore, the integration of 3DE into clinical practice is hampered by the time-consuming nature of the analysis [11]. Although automated algorithms have been developed to streamline the process, manual corrections are often required, particularly in patients with complex cardiac anatomies or poor image quality [2]. This manual intervention not only increases the time required for analysis but also introduces additional variability, as the extent of corrections can vary significantly between operators [14].

Given these challenges, there is a pressing need for universal guidelines that standardize the use of 3DE in clinical practice [4]. Such guidelines should encompass all aspects of 3DE, from image acquisition and analysis to reporting and interpretation [8]. Standardized protocols for image acquisition would help ensure consistent quality across different settings, while guidelines for post-processing would reduce variability in measurements and improve interobserver agreement [2]. Moreover, these guidelines should include recommendations for the use of automated analysis tools, specifying when and how manual corrections should be applied [12]. The clear, evidence-based protocols, would help minimize operator dependence and ensure that 3DE measurements are reliable and reproducible, regardless of the operator’s experience or the equipment used [3]. In addition to technical guidelines, it is also important to establish standardized reference values for 3DE measurements [4]. Currently, reference values for cardiac volumes and function are often based on 2DE data, which may not be directly applicable to 3DE [2].

The development of large, multi-center databases that collect 3DE data from diverse populations would be invaluable in establishing these reference values, ensuring that 3DE measurements can be accurately interpreted in clinical practice [12].

To transcend this barrier, universal guidelines are required to standardize all issues related to 3DE image acquisition and interpretation. these current challenges and providing at least some evidence-based clear-cut protocols will make 3DE an independent, reliable tool in modern cardiology.

## Conflict of Interest

The authors declare that they have no competing interests.
